# Nesidioblastosis and Insulinoma: A Rare Coexistence and a Therapeutic Challenge

**DOI:** 10.3389/fendo.2020.00010

**Published:** 2020-01-24

**Authors:** Angela Dardano, Giuseppe Daniele, Roberto Lupi, Niccolò Napoli, Daniela Campani, Ugo Boggi, Stefano Del Prato, Roberto Miccoli

**Affiliations:** ^1^Section of Diabetes and Metabolic Disease, Department of Clinical and Experimental Medicine, University of Pisa, Pisa, Italy; ^2^Division of General and Transplant Surgery, University of Pisa, Pisa, Italy; ^3^Division of Pathology, University of Pisa, Pisa, Italy

**Keywords:** persistent hyperinsulinemic hypoglycemia, adult-onset β-cell nesidioblastosis, insulinoma, surgery, management

## Abstract

**Background:** Nesidioblastosis and insulinoma are disorders of the endocrine pancreas causing endogenous hyperinsulinemic hypoglycemia. Their coexistence is very unusual and treatment represents a still unresolved dilemma.

**Case Description:** The patient was a 43-year-old Caucasian woman, with a 2-year history of repeated severe hypoglycemic events. The diagnostic work-up was strongly suggestive of insulinoma and the patient was submitted to surgical treatment carried out laparoscopically under robotic assistance. However, surgical exploration and intraoperative ultrasonography failed to detect a pancreatic tumor. Resection was therefore carried out based on the results of selective intra-arterial calcium stimulation test, following a step-up approach, eventually leading to a pancreatoduodenectomy at the splenic artery. The histopathology examination and the immunohistochemical staining were consistent with adult-onset nesidioblastosis. After surgery, the patient continued to experience hypoglycemia with futile response to medical treatments (octreotide, calcium antagonists, diazoxide, and prednisone). Following multidisciplinary evaluation and critical review of a repeat abdominal computed tomography scan, a small nodular lesion was identified in the tail of the pancreas. The nodule was enucleated laparoscopically and the pathological examination revealed an insulinoma. In spite of the insulinoma resection, glycemic values were only partially restored, with residual nocturnal hypoglycemia. Administration of uncooked cornstarch (1.25 g/kg body weight) at bedtime was associated with significant improvement of interstitial glucose levels (*p* < 0.0001) and reduction of nocturnal hypoglycemia episodes (*p* = 0.0002).

**Conclusions:** This report describes a rare coexistence of adult-onset nesidioblastosis and insulinoma, suggesting the existence of a wide and continuous spectrum of proliferative β-cell changes. Moreover, we propose that uncooked cornstarch may offer an additional approach to alleviate the hypoglycemic episodes when surgery is impracticable/unaccepted.

## Introduction

Nesidioblastosis and insulinoma represent the main cause of endogenous hyperinsulinemic hypoglycemia in infants and in apparently healthy adults, respectively (1). Adult-onset nesidioblastosis is very rare (2, 3) and its cause is still unknown. The combination of nesidioblastosis and insulinoma is clinically relevant as each of these pathological entities is rare. We herein describe an unusual case of adult-onset nesidioblastosis coexisting with an insulin-secreting pancreatic adenoma. We also report our preliminary experience on the management of persistent hyperinsulinemic hypoglycemia with uncooked cornstarch.

## Case Report

**Figure 1:**
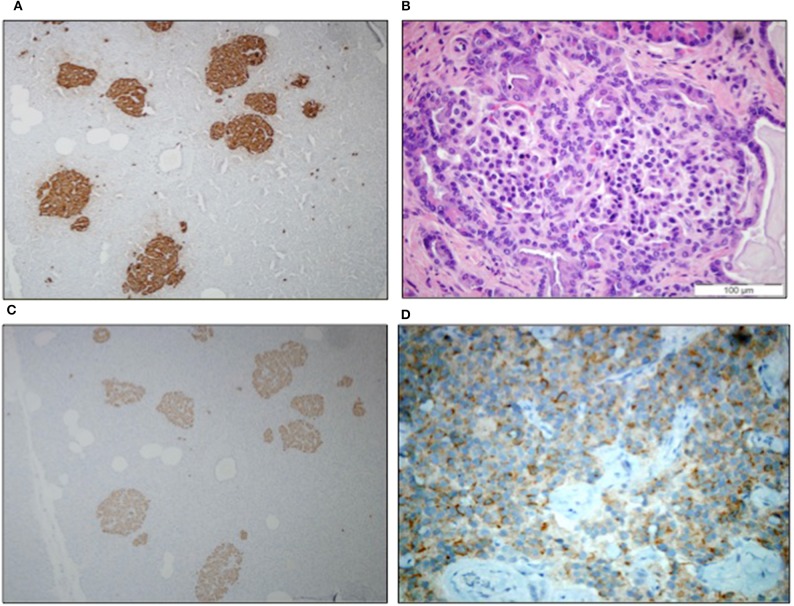
**(A)** Immunostaining of chromogranin highlighted the enlarged and irregular islets (10x); **(B)** ductuloinsular complexes haematoxylin-eosin staining (40x); **(C)** islets with diffuse positivity for insulin (10x); and **(D)** neuroendocrine tumor immunoreactive for insulin (10x).

A 43-year-old Caucasian woman was referred to our Clinic because of a history of repeated hypoglycemic episodes fulfilling the Whipple's triad. The lady reported an initial episode of difficult wakening up associated with temporary amnesia that resolved with carbohydrate intake. Afterwards, the patient experienced frequent episodes of hypoglycemia with prevalence of neuroglycopenic symptoms. Such episodes occurred both in fasting as well as post-prandial state and were relieved by the intake of simple and complex carbohydrates. Capillary blood glucose measurements at the time of these episodes were often <2.78 mmol/L. There was no family history of diabetes mellitus and the patient did not smoke or drink alcohol. On admission, physical examination revealed class I obesity (31 kg/m^2^). Fasting plasma glucose and hemoglobin A1c values were 2.94 mmol/L and 26 mmol/M, respectively. The results of the laboratory workup for rare forms of hypoglycemia were all within the normal range. No sulfonylureas were detected in the urine and search of serum anti-insulin and anti-insulin receptor antibodies was negative. During a consecutive 72-h fasting, plasma glucose reached 2 mmol/L after 18 h, with no suppression of plasma insulin (82.6 pmol/L) and C-peptide concentrations (1.7 nmol/L), compatible with autonomous insulin secretion. Both an abdominal ultrasound and high-resolution contrast-enhanced computed tomography scan were negative for insulinoma and an indium^111^-octreotide scan did not show focal abnormalities. Magnetic resonance imaging examination was not performed due to claustrophobia. ^68^Ga somatostatin receptor PET/CT and glucagon-like peptide-1 (GLP-1) receptor scintigraphy were not performed as they were not available in our Center. The patient then underwent a selective catheterization of the gastroduodenal, splenic, and superior mesenteric arteries with selective arterial calcium stimulation (calcium gluconate 0.025 mEq/kg body weight) and hepatic venous sampling for insulin determination (4). A selective positive response of inappropriate insulin secretion in the region of the gastroduodenal artery was suggestive for an insulinoma of the head/neck of the pancreas (Table 1). Because of the recurrent severe hypoglycemic episodes and the results of calcium stimulation test, the patient was scheduled for robotic assisted surgery in March 2015. Surgical exploration and intraoperative ultrasonography with a dedicated endoscopic ultrasound probe failed to identify a pancreatic tumor. Based on the results of calcium stimulation test, the pancreatic neck was resected first and sent for frozen section evaluation. As no tumor was identified by frozen section, resection was extended a couple of centimeters to the left. As frozen section histology showed again no tumor and considering that the results of calcium stimulation test indicated the tail of the pancreas as the site at the lowest probability to hide the insulinoma, the surgical procedure was completed by removing the head of the pancreas (i.e., pancreatoduodenectomy at the splenic artery). Because of the small size of the pancreatic duct and the extremely soft consistency of the pancreatic stump coupled with well-evident fatty infiltration, a pancreatic anastomosis was not performed, and a controlled external fistula was instead created by inserting a small catheter into the pancreatic duct. Macroscopic examination revealed regular architecture of the pancreas head measuring 4 × 3.5 × 3.5 cm; no tumor was identified. Microscopic examination showed a diffuse increase in islet tissue forming islets of various size (Figure 1A). In the same area, small and medium sized islets were associated with proliferating pancreatic ductules, forming ductuloinsular complexes (Figure 1B). The majority of medium and large sized islets had irregular margins. Immunohistochemistry revealed that the hyperplastic islets were positive for chromogranin A and synaptophysin; glucagon was evidenced in the periphery of the islets and insulin was positive in the majority of islet cells (Figure 1C). The morphologic and immunohistochemical findings were consistent with adult-onset nesidioblastosis. In spite of the surgery, there was no reduction of circulating plasma levels of insulin with persistence of the hypoglycemic episodes requiring during hospitalization parenteral glucose infusion and parenteral nutrition. Treatments with somatostatin analogs and calcium antagonists were futile, while diazoxide was withdrawn due to gastrointestinal intolerance and severe fluid retention. A slight reduction of the severity of hypoglycemia was obtained only with prednisone (40 mg daily). Because of recurrent episodes of infections, related to the presence of external pancreatic fistula, and the occurrence of new episodes of severe hypoglycemia, the option of a second surgery, including completion pancreatectomy, was properly discussed with the patient. In preparation for the second surgery, patient repeated an abdominal computed tomography scan that detected 1 cm nodular lesion in the pancreatic tail far from the pancreatic duct. In January 2017 the patient underwent laparoscopic enucleation of the small pancreatic tumor. During the same procedure the external pancreatic fistula was converted into a pancreatogastrostomy. Frozen sections and subsequent histology documented a well-differentiated, insulin-secreting neuroendocrine tumor, grade 1, measuring 1 × 0.6 × 0.4 cm (Figure 1D). Although the incidence of insulinoma in multiple endocrine neoplasia type 1 (MEN-1) is relatively uncommon (5, 6), an endocrine work-up was also pursued. She had no family history of insulinoma or MEN-1; her PTH level was 4.24 pmol/L (normal range, 0.84–4.24 pmol/L), IGF-1 level 15.4 nmol/L (normal range, 7.0–26 nmol/L), PRL level 13.16 μg/L (normal range, 2–25 μg/L), cortisol level 356.04 nmol/L (normal range, 184.92–623.76 nmol/L), ACTH level 3.74 pmol/L (normal range, <11 pmol/L), and calcium level was 2.24 mmol/L (normal range, 2.1–2.5 mmol/L). To this date, the patient did not wish to perform any other test, but a possible genetic analysis will be discussed in the future. Upon discharge, the patient was equipped with the FreeStyle Libre Flash Glucose Monitoring System (Abbot Diabetes Care, Alameda, California, USA) that documented recurrent nocturnal hypoglycemia. Coincidentally with hypoglycemic episodes, plasma insulin concentrations were 71.3 pmol/L and C-peptide 1.15 nmol/L. In the attempt to reduce the rate and severity of hypoglycemia uncooked cornstarch (1.25 g/kg body weight) was administered at bedtime. After 14 day of uncooked cornstarch supplementation, interstitial glucose levels increased from 4.6 ± 1.83 mmol/L to 5.3 ± 1.62 mmol/L (*p* < 0.0001) along with remarkable reduction of nocturnal hypoglycemia episodes (i.e., glucose ≤ 2.78 mmol/L from 8 p.m. to 8 a.m.; *p* = 0.0002). The uncooked cornstarch was well-tolerated with no gastrointestinal side effects. As the patient was hesitant toward any further surgical procedure, she was closely monitored. At 1-year follow-up, no pancreatic exocrine insufficiency or diabetes was observed, and a high definition contrast enhanced abdominal computed tomography showed a normal residual pancreatic tissue.

**Table 1:** Results of selective intra-arterial calcium injection of the major pancreatic arteries with hepatic venous sampling.

	**Superior mesenteric artery**			**Splenic artery**			**Gastroduodenal artery**		
**Time** **(seconds)**	**Glucose** **(mmol/L)**	**Insulin** **(pmol/L)**	**C-peptide** **(nmol/L)**	**Glucose** **(mmol/L)**	**Insulin** **(pmol/L)**	**C-peptide** **(nmol/L)**	**Glucose** **(mmol/L)**	**Insulin** **(pmol/L)**	**C-peptide** **(nmol/L)**
0	5.5	373.6	2.8	5.4	68.7	2.6	8.3	333.3	4.9
30	9.4	305.5	3.1	8.4	86.8	1.6	4.9	1301.4	8.4
60	6.6	231.2	1.5	6.6	76.4	2.1	4.1	7363.2	6.7
120	8.1	300.0	3.2	6.8	98.6	2.2	6.4	3872.9	6.8

### Methods

Plasma glucose was measured by the glucose oxidase reaction (Glucose Oxidase Analyzer). Plasma insulin and C-peptide were measured by a radioimmunoassay (PANTEC srl Turin, Italy). Cortisol was measured by chemiluminescent Immuno Assays (CLIA). PTH, ACTH, IGF-1, and PRL levels were measured by immunochemiluminometric assay (ICMA). Calcium level was measured by standard method.

## Discussion

Insulinoma is the most frequent cause of hyperinsulinemic hypoglycemia in adults (1) while adult-onset nesidioblastosis accounts for only 0.5–5% of all cases of hyperinsulinemic hypoglycemia in adulthood (2, 3). The pathogenesis of adult-onset nesidioblastosis is unclear, and several mechanisms have been postulated, including dysregulation of β-cell function (7), increased production of growth factors and/or expression of their receptors (8) as well as unidentified genetic variants (9). Nesidioblastosis has been also claimed to be caused by increased levels of β-cell trophic factors in subjects undergoing bariatric surgery (10).

Here we describe an unusual case of adult-onset nesidioblastosis coexisting with an insulin-secreting pancreatic adenoma. The association of these two entities is a rare clinical finding. To the best of our knowledge, the association between nesidioblastosis and neoplastic diseases of the endocrine pancreas has been described in just a few cases (11–21). The peculiarity of our case is that the diagnoses of nesidioblastosis and insulinoma were not concomitant. Though we cannot rule out that early diagnostic procedures may have failed identifying the pancreatic neoplasia, our case suggests that the two conditions may arise at different time accounting for a wide and continuous spectrum of proliferative β-cell changes. In keeping with this view, a progression from nesidioblastosis to pancreatic tumor has been observed in experimental models (22). Moreover, induction of nesidioblastosis can enhance pancreatic carcinogenesis (23), and a spectrum of proliferation from nesidioblastosis to islet cell hyperplasia to multiple tumors to metastases has previously been reported (24). An insulinoma relapse on the background of a nesidioblastosis has been recently described (25), although identification of molecular mechanisms underlying this transition still remains to be established. Our case is another example of how complex it can be to differentiate an insulinoma from nesidioblastosis as the clinical presentation can be similar, and imaging studies inconclusive. Adult-onset nesidioblastosis is more commonly characterized by post-prandial hypoglycemia (2). In our case, symptoms developed both in the absorptive and post-absorptive state though positive fasting test was supportive of the diagnosis of insulinoma. The overlap of these clinical features made the diagnosis more difficult and uncertain. Imaging studies are generally negative in nesidioblastosis, but insulinomas can be too small to be detected (26). New non-invasive options such as ^68^Ga somatostatin receptor PET/CT and the more recent GLP-1 scintigraphy could have been useful to confirm the localization of the suspected insulinoma. PET/CT scanning with ^68^Ga labeled somatostatin analogs, however, is only positive in 25–31% of insulinoma patients (27). Scintigraphy with radiolabeled GLP-1 receptor analogs has been proposed as a second-line imaging option (28) because insulinomas frequently overexpress this receptor (29, 30) and its performance could offer an opportunity (6). Unfortunately, this method is not available everywhere. In our case, the patient should have performed the test and she refused to be moved away. Nonetheless the ability of this procedure to discriminate a focal insulinoma from diffuse nesidioblastosis has not been validated yet. When pre-operative imaging studies are negative, the intra-arterial calcium stimulation test is recommended (31). If the test allows regionalization, the diagnostic algorithm suggests confirming the localization by intraoperative methods. If localization is not achieved, a pancreatic tail resection for histologic evaluation is recommended (31). Our patient underwent an intra-arterial calcium stimulation test showing extremely high insulin release at the neck region, very high at the pancreatic body level, high at the pancreatic head, and low/normal at the tail region. The insulin gradient indicated the tail of the pancreas as the site with the lowest probability to host the insulinoma and a decision to resect the pancreatic neck was made, as a deviation from current recommendation, because of the suggestive very high insulin release from that region. Obviously, we could not exclude the possibility of a focal nesidioblastosis generating a gradient across selective arterial vessel stimulation, mimicking firing of a local insulin secreting adenoma, while detection of diffuse insulin response during selective arterial stimulation is more suggestive of nesidioblastosis (32).

Our case is instrumental in highlightening difficulties of the treatment of nesidioblastosis. While the gold-standard therapy for insulinoma is its surgical enucleation, that for nesidioblastosis is controversial. Therapeutic options include low-carbohydrate diet in combination with drugs such as diazoxide, octreotide, steroids, or nifedipine as it has been reported in isolated case reports. Pasireotide, a somatostatin analog used for treatment of acromegaly in adults, has been recently used with some success, though treatment protocol is not yet standardized (25, 33). Nonetheless the majority of the patients with nesidioblastosis undergo surgery though the degree of resection is still a matter of debate. Indeed, there is a very fine balance between removing too much or to little pancreatic tissue as too little may not be associated with significant improvement of the hypoglycemic syndrome and too much may cause pancreatogenic diabetes. Current literature supports a 60–89% resection of the pancreas (i.e., distal or subtotal pancreatectomy) with a >10% risk of subsequent diabetes and a 70% success rate of achieving normoglycemia (34). Our patient initially underwent pancreatoduodenectomy at the splenic artery and, 2 years later, to enucleation of an insulinoma in the pancreatic remnant. She did not develop diabetes, but she continued suffering of low plasma glucose levels. The patient was hesitant toward any further surgical procedure and common medical therapy was either ineffective or associated with side effects, but an improvement was obtained with the use of uncooked cornstarch administered at bedtime. In the early 1980s, uncooked cornstarch was introduced as an alternative to continuous nocturnal nasogastric glucose and frequent daytime feedings for maintaining normoglycemia in glycogen storage disease. This approach is still the mainstay of therapy (35–37). Uncooked cornstarch is a low-glycemic index carbohydrate characterized by slow intestinal degradation and absorption. It takes 4–6 h for the cornstarch to be completely digested. As a source of slowly releasable carbohydrates, uncooked cornstarch produces low glucose peaks and maintains blood glucose stable during fasting. It is given intermittently to provide stable blood glucose concentrations, reducing swings of insulin secretion (35, 36). Based on these properties, the effectiveness of uncooked cornstarch in preventing hypoglycaemia has been demonstrated in several conditions (37–39), including type 1 and type 2 diabetes (40, 41). Uncooked cornstarch has been used in few cases of children with nesidioblastosis (42), but its clinical efficacy in adult-onset nesidioblastosis was not previously assessed. Fourteen days after beginning bedtime cornstarch combined with personalized diet, our patient showed a remarkable reduction of nocturnal hypoglycemia episodes and an increase in glucose levels, without any relevant side effect.

In conclusion, our case report shows that: (1) nesidioblastosis and insulinoma can occur in the same patient; (2) preoperative diagnosis of both conditions is difficult, but, if diagnosis is established, it may have much influence on treatment choice; (3) uncooked cornstarch can be considered in patients with persistent hypoglycemia particularly when surgery is impracticable/unaccepted and pharmacologic therapy is ineffective.

## Data Availability Statement

All datasets generated for this study are included in the article/supplementary material.

## Ethics Statement

Written informed consent was obtained from the individual(s) for the publication of any potentially identifiable images or data included in this article.

## Author Contributions

AD collected the data and wrote the manuscript with the support of GD and RM. SDP and UB overviewed the discussion. DC analyzed the specimen and provided the pictures. NN reviewed CT scan. RL performed lab measurements. All authors discussed the case and approved the final manuscript.

### Conflict of Interest

The authors declare that the research was conducted in the absence of any commercial or financial relationships that could be construed as a potential conflict of interest.
